# Automated Segmentation of Optical Coherence Tomography Angiography Images: Benchmark Data and Clinically Relevant Metrics

**DOI:** 10.1167/tvst.9.13.5

**Published:** 2020-12-03

**Authors:** Ylenia Giarratano, Eleonora Bianchi, Calum Gray, Andrew Morris, Tom MacGillivray, Baljean Dhillon, Miguel O. Bernabeu

**Affiliations:** 1 Usher Institute, University of Edinburgh, Edinburgh, UK; 2 Princess Alexandra Eye Pavilion, NHS Lothian, Edinburgh, UK; 3 Edinburgh Imaging, University of Edinburgh, Edinburgh, UK; 4 Health Data Research UK, London, UK; 5 Centre for Clinical Brain Sciences, University of Edinburgh, Edinburgh, UK; 6 School of Clinical Sciences, University of Edinburgh, Edinburgh, UK

**Keywords:** optical coherence tomography angiography, automated segmentation, retinal vasculature

## Abstract

**Purpose:**

To generate the first open dataset of retinal parafoveal optical coherence tomography angiography (OCTA) images with associated ground truth manual segmentations, and to establish a standard for OCTA image segmentation by surveying a broad range of state-of-the-art vessel enhancement and binarization procedures.

**Methods:**

Handcrafted filters and neural network architectures were used to perform vessel enhancement. Thresholding methods and machine learning approaches were applied to obtain the final binarization. Evaluation was performed by using pixelwise metrics and newly proposed topological metrics. Finally, we compare the error in the computation of clinically relevant vascular network metrics (e.g., foveal avascular zone area and vessel density) across segmentation methods.

**Results:**

Our results show that, for the set of images considered, deep learning architectures (U-Net and CS-Net) achieve the best performance (Dice = 0.89). For applications where manually segmented data are not available to retrain these approaches, our findings suggest that optimally oriented flux (OOF) is the best handcrafted filter (Dice = 0.86). Moreover, our results show up to 25% differences in vessel density accuracy depending on the segmentation method used.

**Conclusions:**

In this study, we derive and validate the first open dataset of retinal parafoveal OCTA images with associated ground truth manual segmentations. Our findings should be taken into account when comparing the results of clinical studies and performing meta-analyses. Finally, we release our data and source code to support standardization efforts in OCTA image segmentation.

**Translational Relevance:**

This work establishes a standard for OCTA retinal image segmentation and introduces the importance of evaluating segmentation performance in terms of clinically relevant metrics.

## Introduction

A number of studies have demonstrated that phenotypes of the retinal vasculature represent important biomarkers for early identification of pathologic conditions such as diabetic retinopathy,^1^ cardiovascular disease,^2^ and neurodegenerative disease.^3^ Therefore information regarding structural and functional changes in the retinal blood vessels can play a crucial role in the diagnosis and monitoring of these diseases.

Optical coherence tomography angiography (OCTA) is a novel noninvasive imaging modality that allows visualization of the microvasculature in vivo across retinal layers. It is based on the principle of repeating multiple OCT B-scans in rapid succession at each location on the retina. Static tissues will remain the same, whereas tissues containing flowing blood cells will show intensity variations over time. OCTA can provide angiograms at different retinal depths and, unlike fluorescein angiography, does not require any dye injection, which may carry the risk of adverse reactions.^4^ OCTA diagnosis potential has already been established in the context of neurovascular disease, diabetes mellitus before development of retinopathy, and, more recently, in chronic kidney disease (CKD). In Yoon et al.,^5^ microvascular characteristics calculated from OCTA images are compared between Alzheimer's disease patients, mild cognitive impairment (MCI) patients, and cognitively intact controls. Results showed a decrease in vessel density (VD) and perfusion density (PD) of Alzheimer participants compared with the MCI and controls, opening to the possibility that changes in the retinal microvasculature may mirror small vessel disease in the brain, which is currently not possible to image clinically.

Multiple studies on diabetic retinopathy have demonstrated that measurements from the foveal avascular zone (FAZ), for example, area and acircularity, in OCTA images are discriminant features in diabetic eyes compared to healthy individuals, even before retinopathy develops.^6^^,^^7^ Finally, a recent study on renal impairment^8^ demonstrated the potential of OCTA to find associations between changes in the retina and CKD. OCTA scans revealed a close association between CKD and lower paracentral retinal vascular density in hypertensive patients.

Measurements used in these studies are based on quantifying phenotypes such as vessel density (VD), fractal dimension (FD), and percentage area of nonperfusion (PAN), extracted from binary masks of OCTA images.^9^^,^^10^ However, the accuracy of these measurements and their reproducibility relies on the quality of the image segmentation. Because manual segmentation of blood vessels is a time-consuming procedure that requires interrater and intrarater repeatability, there is a necessity to establish a fast automated method not affected by individual subjectivity. The development of automated segmentation algorithms for OCTA images is a novel research field and no consensus exists in the literature about the best approaches. For example, in Alibhai et al.^11^ and Krawitz et al.,^12^ OCTA phenotypes are calculated on manually traced vessels. Simple thresholding procedures are used in Nesper et al.,^9^ Onishi et al.,^13^ and Hwang et al.^14^ Hessian filters followed by thresholding are applied to the original image to enhance vessels structure in Kim et al.^15^ and Zhang et al.^16^ Frame averaging to enhance vessels has been proposed in Schmidt et al.^17^ before applying Sobel filter, hysteresis thresholding method, and opening and closing procedures for FAZ detection. In Jesus et al.,^18^ circumpapillary microvascular density (cpmVD) is computed without the use of a segmentation method. The annular area around the optic disc was converted into a rectangular shape region, and a third-order median filter was applied to the vector representing column means of that region. Finally, a spline over the local maxima is used to estimate the value of the cpmVD. A convolutional deep neural network approach was proposed in Prentašic et al.,^19^ and more recently U-Net and CS-Net architectures were adapted to OCTA in Mou et al.^20^ However, how these different approaches compare to each other is not known. Furthermore, it is currently unknown how these methods perform when it comes to preserving network connectivity in the segmentation. This is a key aspect that can enable advanced vascular network phenotyping based on network science approaches.^21^^,^^22^

In this work, we take advantage of OCTA images from the PREVENT cohort https://preventdementia.co.uk/, an ongoing prospective study aimed to predict early onset of dementia.^23^ Previous studies have shown OCTA imaging as a source of biomarkers for neurodegenerative disease,^24^^,^^25^ and together with MRI scans, OCTA images are being investigated in PREVENT. We derive and validate the first open dataset of retinal parafoveal OCTA images with associated ground truth manual segmentations. Furthermore, we establish a standard for OCTA image segmentation by surveying a broad range of state-of-the-art vessel enhancement and binarization procedures. We provide the most comprehensive comparison of these methods under a unified framework to date. Furthermore, we report on the importance of preserving full network connectivity in the segmentation of angiograms to enable deep vascular phenotyping and introduce two new network structure evaluation metrics: the largest connected component ratio (LCC) and the topological similarity score (TopS). Our results show that, for the set of images considered, the U-Net and CS-Net architecture achieve the best performance in Dice score (both 0.89), but the latter reaches a better performance in TopS. Among the handcrafted filter enhancement methods from those considered, optimally oriented flux is the best in both pixelwise and network metrics. Our results demonstrate that methods with equal Dice score (e.g., adaptive thresholding and OOF) can perform substantially different in terms of LCC or TopS. Furthermore, we compare the relative error in the computation of clinically relevant vascular network metrics (e.g., foveal avascular zone area and vessel density) across segmentation methods. Our results show up to 25% differences in vessel density and 24% in FAZ area depending on the method employed and that U-Net outperforms all other methods when investigating the FAZ. These findings should be considered when comparing the results of clinical studies and performing meta-analyses. Finally, we release our data and source code to support standardization efforts in OCTA image segmentation.

## Methods

### Data Acquisition and Manual Segmentation

**Figure 1.**
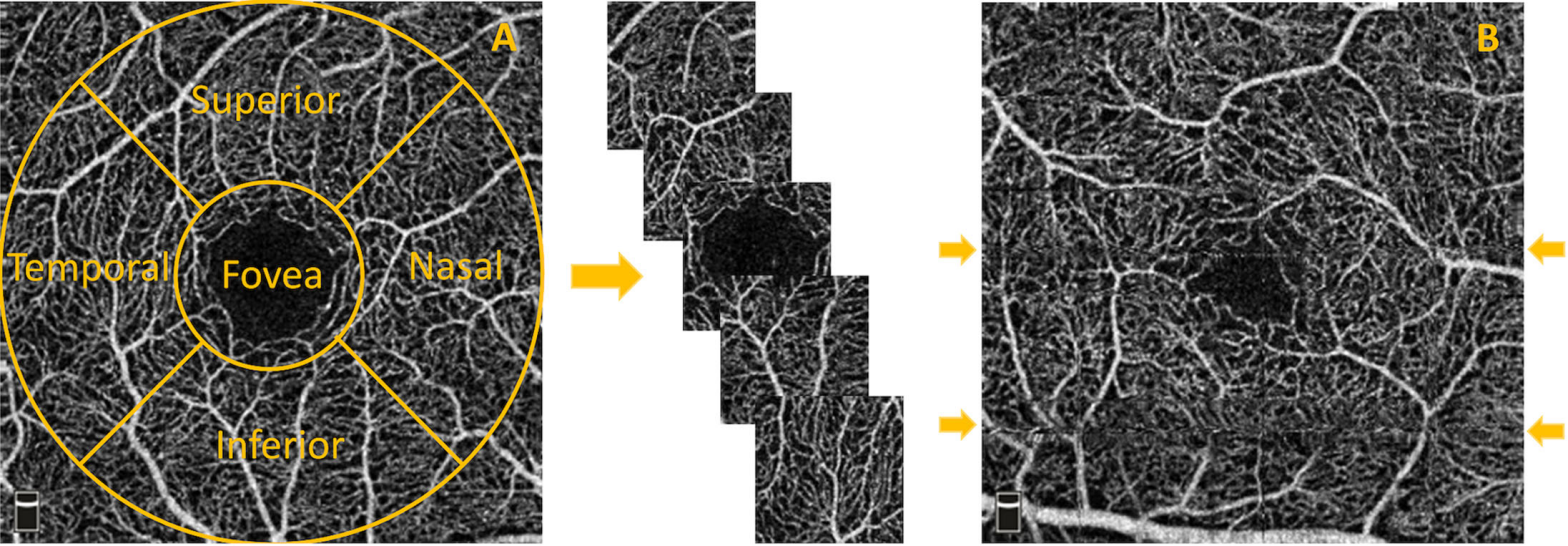
(A) Extraction of images from each clinical region of interest: superior, nasal, foveal, inferior, and temporal. (B) Examples (*arrows*) of horizontal artifacts in OCTA images.

Imaging was performed using the commercial RTVue-XR Avanti OCT system (OptoVue, Fremont, CA). Consequent B-scans, each one consisting of 304 × 304 A-scans, were generated in 3 × 3 mm and 6 × 6 mm fields of view centered at the fovea. Maximum decorrelation value is used to generate en face angiograms of superficial, deep, and choriocapillaris layers. In this work, we selected images only of the superficial layer (containing the vasculature enclosed in the internal limiting membrane layer (ILM) and the inner plexiform layer (IPL)) with 3 × 3 mm field of view from left and right eyes of participants with and without family history of dementia as part of a prospective study aimed to find early biomarkers of neurodegenerative diseases (PREVENT). From the initial 17 participants, we extracted five subimages, one from each clinical region of interest (ROI): superior, nasal, inferior, temporal, and foveal (Fig. 1A), and a target of 55 ROIs among the best quality images was set for the purpose of this study. Criteria for the exclusion were based on major visible artifacts, such as very poor signal to noise ratio, stretching and quilting defects.^26^ The final 55 ROIs were selected from 11 participants (nine females and two males, aged 44–59, not presenting any ocular disease), and split into training (30 ROIs) and test (25 ROIs). Two foveal regions in the training set presented fragmented avascular zone.^27^

### Manual Segmentation

A number of challenges need to be overcome in OCTA manual segmentation: images suffer from poor contrast, low signal to noise ratio and can contain motion artifacts generated during the scan acquisition. The most common visible artifacts are vertical and horizontal line distortions, as shown in Figure 1B. Furthermore, the fact that images are constructed from the average of a volume means that, in our segmentation, we cannot distinguish vessels going past each other at different depths. In general, bigger vessels appear brighter and easier to trace; however, the smallest capillaries are challenging to segment and therefore are affected to subjective interpretation by any given rater.

Previous OCTA studies have performed manual continuous blood vessel delineation with or without consideration of vessel width.^19^^,^^20^ Given the sources of uncertainty previously described, this approach may overinterpret vessel connectivity and suffer from reproducibility issues that remain currently unexplored in the literature. Instead, we adopted a more conservative approach and performed pixelwise manual segmentation selecting all pixels enclosed in the vasculature (using the ITK-SNAP software).^28^ A previous study performing pixelwise segmentation^29^ did not assess the reproducibility of the segmentations and could not resolve the finest capillaries in the scans.

### Automated Image Segmentation Methods

Vessel enhancement approaches consist of filters that improve the contrast between vessels and background. We chose four well-known handcrafted filters for blood vessel segmentation, based on implementation availability and previous applications to the enhancement of tubular-like structures in retinal images: Frangi,^30^ Gabor,^31^ SCIRD-TS,^32^ and OOF.^33^ All of these filters require parameter tuning. In our case, from a range of possible configurations, we selected the optimal set of parameters that gave the best performance when compared to the manual segmentation (see Supplementary Table S1).

Although handcrafted filters work in many cases, often real images do not satisfy their assumptions (e.g., locally tubular structure and gaussian intensity profile). To overcome this issue, probabilistic and machine learning frameworks have been proposed.^29^^,^^19^ In this study, we considered the latter by adopting three deep learning architectures. We used a pixelwise convolutional neural network (CNN), U-Net, and the more recently proposed CS-Net.^20^ The design of the CNN for pixelwise classification is based on the one proposed in Prentašic et al.^19^ for OCTA segmentation. It consists of three convolutional layers with rectified linear unit activation (ReLU), each followed by maxpooling. To reduce the risk of overfitting, dropout is used before the last fully connected layer. Cross-entropy and adam optimizer were used during the learning process. For each training image we randomly extracted the same number of vessel and background pixels to balance the classes. A patch containing the pixel to classify and its 61 × 61 neighborhood is used as input to the network. More than 200,000 patches were used during the training. Finally, the probability of belonging to a vessel or background is then used to generate the enhanced grayscale image (see Supplementary Table S2).

Developed for biomedical image segmentation, U-Net is a fully convolutional neural network characterized by a contracting path and an expansive path that confer to the network its U shape. It has proved to be fast and accurate, even with few training images. The architecture consists of modules of two repeated convolutional layers with ReLU activation function followed by maxpooling for the encoder path, upsampling and two repeated convolutional layers for the decoder path (see Supplementary Table S3). The lowest resolution is 8 × 8 pixels, with binary cross-entropy used as loss function and SGD as optimizer. From each ROI, 1000 patches of size 32 × 32 are extracted to train the network, for a total of 30,000 training inputs.

Finally, the recently proposed CS-Net was tested using the same sub-patches procedure previously described. As U-Net, this architecture is characterized by a contractive and an expansive path. However, between those paths, two other elements are present: the spatial and channel attention module. The first uses spatial correlation to acquire global contextual features; the latter uses changes in intensity across channels to extract features. Adam optimizer and MSE loss are used to train the model. Given our initial sample size, data augmentation (flipping horizontally or vertically) has been used in all the three architectures with the 10% of training inputs used as validation set.

Vessel enhancement is often followed by a threshold step to obtain the vessel binary mask. However, modern methods employ the enhanced vasculature as a preliminary step for more advanced binarization algorithms, such as machine learning (ML) classifiers. In this work we use adaptive thresholding as baseline binarization procedure, a method that takes into account spatial variations in illumination^34^ in a specified neighborhood of the pixel. We compared this approach with other binarization methods, support vector machines (SVMs), random forest (RF), and k-nearest neighbors (k-NN) as a binarization procedure in the case of Frangi, Gabor, SCIRD-TS. A two-step binarization procedure, suggested in Li et al.,^35^ has been used in the case of OOF, a global threshold for larger vessels and adaptive threshold for the smallest ones. Finally, a global thresholding, based on the shape of the pixel intensity histogram, is used to binarize the probability maps obtained from the CNN architecture, adaptive thresholding is applied to the output of U-Net, and the Otsu method^36^ in the case of CS-Net. After all binarization procedures, morphological opening is performed to remove small disconnected pixel structures. In each of the ML binary classifiers, we used seven features to characterize pixels: intensity-based features extracted from a 3 × 3 pixel neighborhood (intensity value, range, average, standard deviation, and entropy) and geometric features (the local curvature information provided by the hessian eigenvalues).^37^

### Segmentation Evaluation

Cohen's kappa coefficient is a robust statistic for testing interrater and intrarater variability.^38^

Considering *Pr(a)* and *Pr(e)* as the observed agreement and the chance agreement, respectively, it can be computed as

(1) $$\kappa =\frac{Pr\left(a\right)\;-\;Pr\left(e\right)}{1-Pr\left(e\right)}.$$

In our study *Pr(a)* is the accuracy in pixel classification (vessel *vs* background) and *Pr(e)* is the sum of the probability of both raters randomly selecting vessel pixels and the probability of both of them randomly selecting background pixels for a given ROI.

For the ROIs in the test set, pixelwise comparison between manual and automated segmentation was performed using accuracy, precision, recall along with Dice similarity coefficient defined as

(2) $$Dice=\frac{2TP}{2TP\;+\;FP\;+\;FN},$$

here TP, FP, FN represent true positive, false positive, and false negative, respectively.

Furthermore, for the evaluation of the global quality of segmentation, we used the CAL metric proposed in Gegundez-Arias.^39^ It is based on three descriptive features:

• connectivity (C), to assess the fragmentation degree between segmentations, described mathematically by the formula
  (3) $$C\left(S,S_{GT}\right)=1-min\left(1,\frac{\left|{\#}_{C}S_{GT}-{\#}_{C}S\right|}{\#S_{GT}}\right),$$
  where *#_C_S* and *#_C_S_GT_* are the number of connected components in the segmented and ground truth image, while *#S_GT_* is the number of vessel pixels in the mask;
• area (A), to evaluate the degree of overlapping, defined as
  (4) $$A\left(S,S_{GT}\right)=\frac{\#(\left(\delta_{\alpha }\left(S\right)\cap S_{GT}\right)\cup \left(S\cap \delta_{\alpha }\left(S_{GT}\right)\right)}{\#\left(S\cup S_{GT}\right)},$$
  where δ_α_ is a morphological dilation using a disc of radius α;
• length (L), to capture the degree of coincidence, described by
  (5) $$\begin{array}{c} \begin{array}{c} L\left(S,S_{GT}\right)=\frac{\#\left(\left(\varphi \left(S\right)\cap \delta_{\beta }S_{GT}\right)\right)\cup \left(\delta_{\beta }\left(S\right)\cap \varphi \left(S_{GT}\right)\right)}{\#\left(\varphi \left(S\right)\cup \varphi \left(S_{GT}\right)\right)}, \end{array}\; \end{array}$$
  where ϕ indicates a skeletonisation procedure and δ_β_ is a morphological dilatation using a disc of radius *β*.

Considering vessel width and closeness between capillaries in OCTA images, we set α and β both equal to 1. The product of C, A, and L, (CAL) results sensitive to the vascular features and takes values in the range [0,1], with the 0 denoting the worst segmentation and 1 the perfect segmentation.

Despite CAL contains a connectivity component, its effect is weighted by the values of area (A) and length (L) metrics. Hence, we introduce a new metric, namely the largest connected component ratio (LCC), with the aim of penalizing those methods that do not retrieve connections of the vascular network. LCC is defined as

(6) $$LCC=1-min\left(1,\frac{\left|\#LCC_{S}-\#LCC_{GT}\right|}{\#LCC_{GT}}\right),$$

where $\#LCC_{S\;}$and $\#LCC_{GT}$ are the lengths, in terms of number of pixels, of the largest connected component in the skeleton of the segmented and ground truth images.

The closest to 1 the LCC ratio is, the more similar is in structure the largest connected component of the segmented image compared to the ground truth. Using LCC in conjunction with Dice score, we provide information about pixelwise similarity and connectivity. Indeed, a single pixel difference does not affect Dice; however, it may drastically change the number of connected components, affecting the LCC ratio.

To evaluate the topological accuracy of segmentations, we use the concept of persistent homology and Betti numbers for angiograms described in Hu et al.,^40^ by introducing the topological similarity score (TopS) defined as

(7) $$TopS=1-min\left(1,\frac{\left|{\beta_{1}}_{S}-{\beta_{1}}_{GT}\right|}{{\beta_{1}}_{GT}}\right),$$

where β_1_ is the first Betti number associated with the image and indicating the counts of one-dimensional holes, we compute the similarity between topological structures.

Finally, popular biomarkers in the literature for OCTA images include vessel density, FAZ area, and FAZ acircularity index. We investigate how the segmentation method affects these metrics by reporting the relative error against ground truth measurements. Vessel density is defined as the number of white pixels over the total number of pixels. To compute FAZ area and acircularity index, we convert the skeleton of the image into a graph object where the largest loop (face) in the network is identified as the FAZ.^41^ This method takes into account a continuous boundary; therefore images with disconnected contour will show greater FAZ area.

## Results

### Interrater and Intrarater Agreements

The ground truth dataset contains 55 ROIs segmented by one rater (rater A). Rater A (Y.G.) segmented 20 images twice to assess the intrarater agreement. Another set of 20 images was segmented by rater B (E.B.) to determine the interrater reliability. Results show good agreements (for each pair *k > 0.7*) with an average of 0.8 for the intrarater agreement and 0.77 between operators, which demonstrates that the proposed approach to segmentation is reproducible.

### Automated Approaches for Pixelwise Classification

**Figure 2.**
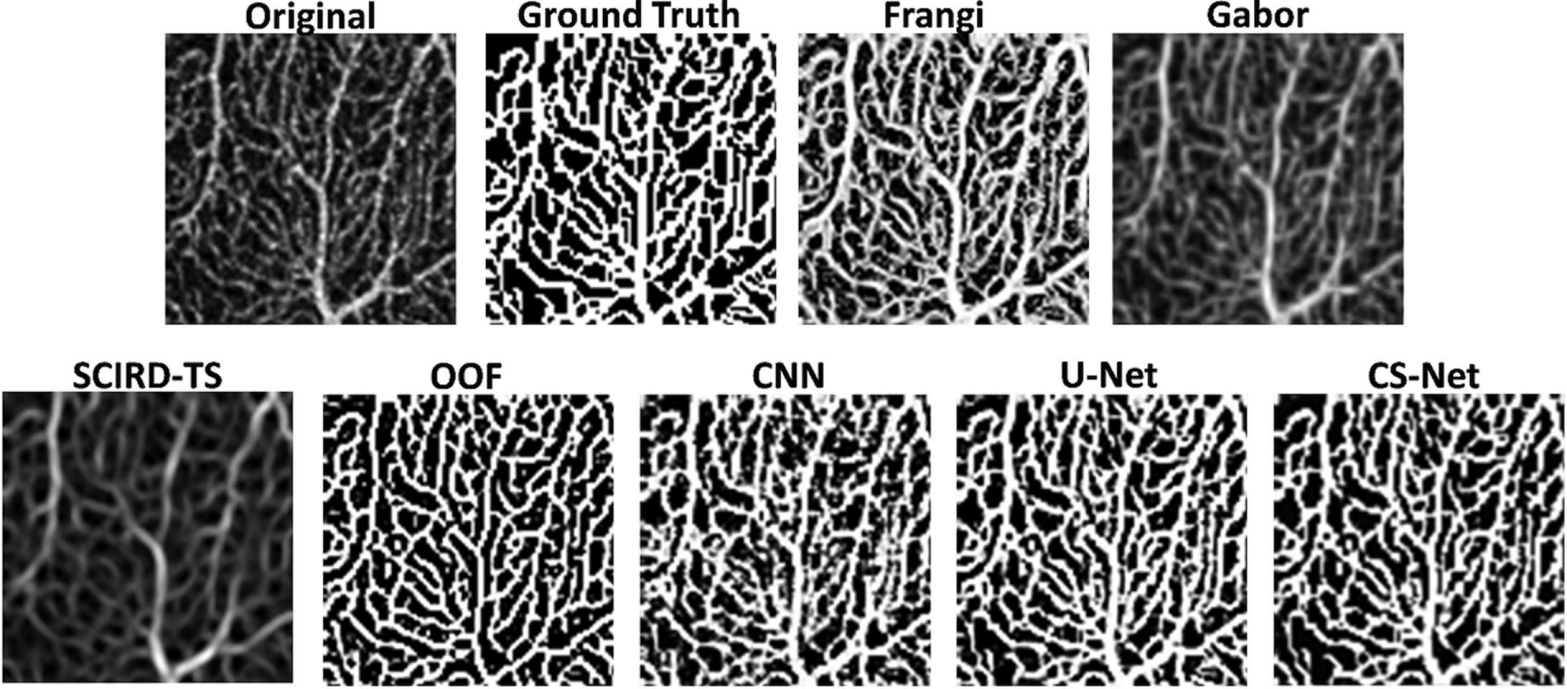
Example of vessel enhancement. Original, ground truth and images after vessel enhancement by using Frangi, Gabor, SCIRD-TS, OOF, CNN, U-Net, CS-Net.

**Figure 3.**
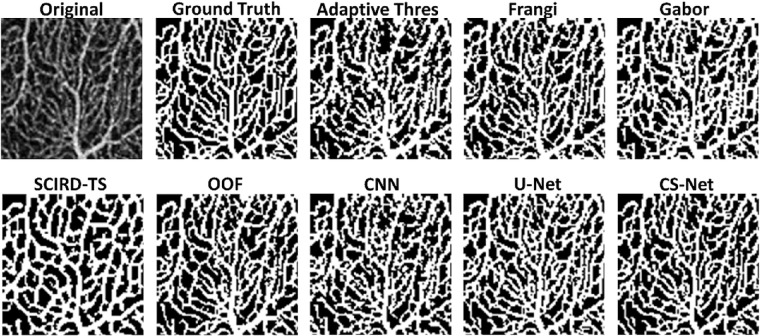
Vessel segmentation in superior parafoveal OCTA image. Original, ground truth images followed by binary images after vessel enhancement by using Frangi (+RF), Gabor (+RF), SCIRD-TS (+SVM), OOF, CNN, U-Net, and CS-Net.

**Figure 4.**
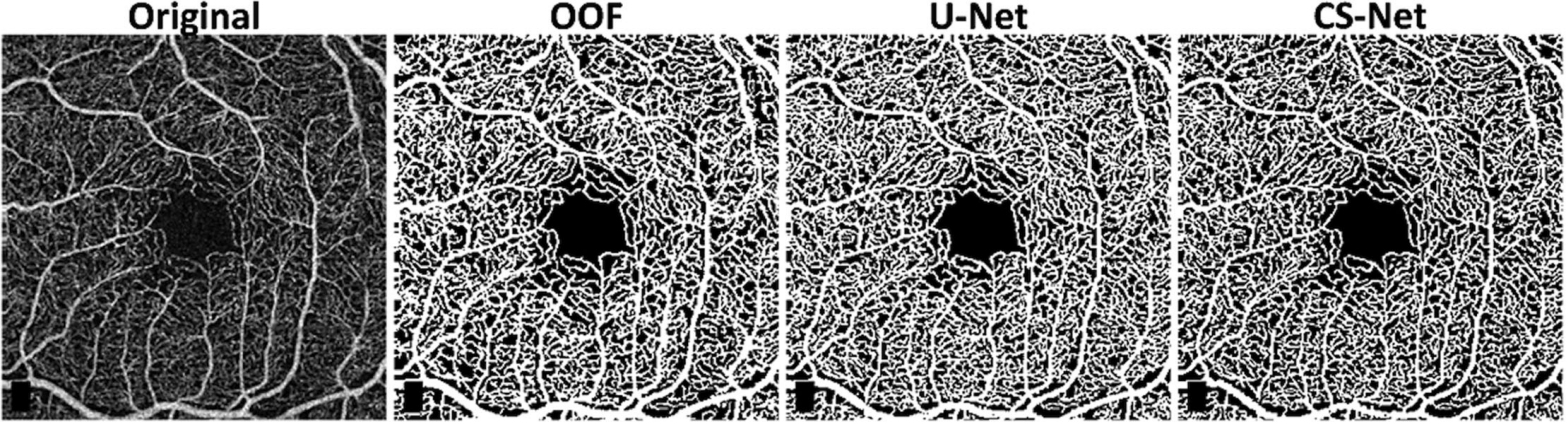
Whole image segmentation by using the best three methods, OOF, U-Net, and CS-Net. Optovue RTVue XR Avanti scan logo on the bottom left corner was removed from the original image.

Segmentation performances according to the metrics proposed are shown in Table 1. U-Net and CS-Net outperform all the other methods, by reaching a Dice score of 0.89. Among the handcrafted filters, OOF and Frangi filters achieve good performances with an average Dice score of 0.86 and 0.85, respectively. Our baseline method, adaptive thresholding without vessel enhancement, achieves comparable Dice performance. However, it encounters difficulties resolving network connectivity as shown by the LCC and TopS metrics compared to Frangi and OOF. The use of machine learning methods as binarization procedure can improve performance compared to thresholding after Frangi, Gabor and SCIRD-TS both in terms of pixelwise and network structure accuracy. Deep learning architectures reach the best results in LCC ratio (CNN, 0.94, U-Net and CS-Net, 0.93) together with the OOF (0.94). The highest TopS score is reached by the CS-Net and OOF, 0.83 and 0.80, respectively. Moreover, the same two methods achieve the two lowest vessel density error (6%, and 10%). Investigating the enhanced images (Fig. 2) we noticed that each method suffers from different deficiencies. Frangi filter clusters nearby vessels, losing important information contained in the microvasculature. Gabor filter enhances centerlines, performing poorly on the detection of vessel edges. SCIRD-TS remodels the vasculature making it more regular and equally spaced. OOF retrieves the smallest capillaries but overenhances noise in the foveal region. Figure 3 shows segmentation results after applying each vessel enhancement method and best binarization procedure. Figure 4 shows whole image segmentations with the best handcrafted and learned filters.

**Table 1.** Segmentation Performances (Best Method Per Column in Bold)

Method	Dice	Acc	Rec	Pre	CAL	LCC	TopS	VD
Adaptive thres (AT)	0.86	0.89	0.89	0.92	0.83	0.83	0.70	14%
Frangi + AT	0.83	0.86	0.83	0.93	0.83	0.88	0.72	21%
Gabor + AT	0.77	0.81	0.78	0.87	0.75	0.76	0.59	24%
SCIRD-TS + AT	0.71	0.76	0.74	0.82	0.66	0.68	0.46	25%
OOF	0.86	0.88	0.87	0.92	0.85	**0.94**	0.80	10%
Frangi + k-NN	0.84	0.87	0.85	0.91	0.86	0.91	0.59	14%
Frangi + SVM	0.85	0.88	0.85	0.93	0.87	0.94	0.76	15%
Frangi + RF	0.85	0.88	0.86	0.92	0.87	0.94	0.75	13%
Gabor + k-NN	0.82	0.84	0.80	0.92	0.84	0.84	0.37	21%
Gabor + SVM	0.83	0.85	0.78	0.94	0.85	0.84	0.45	24%
Gabor + RF	0.83	0.85	0.80	0.93	0.85	0.87	0.45	22%
SCIRD-TS + k-NN	0.72	0.77	0.76	0.82	0.74	0.90	0.35	19%
SCIRD-TS + SVM	0.75	0.79	0.78	0.84	0.75	0.75	0.54	19%
SCIRD-TS + RF	0.74	0.78	0.77	0.83	0.75	0.80	0.65	19%
CNN	0.83	0.86	0.85	0.91	0.85	**0.94**	0.70	14%
U-Net	**0.89**	**0.91**	0.87	**0.97**	**0.90**	0.93	0.67	17%
CS-Net	**0.89**	**0.91**	**0.91**	0.93	**0.90**	0.93	**0.83**	**6%**

### Foveal Avascular Zone

Foveal images are characterized by the presence of a predominantly dark area free from blood vessels called the *foveal avascular zone* (FAZ). We noticed that handcrafted filters have difficulties with those images, overenhancing noise in the central region (Supplementary Fig. S1A). This can lead to vessel detection in the FAZ when segmentation by simple thresholding is applied. Machine learning methods were less affected by this issue since they learned from the ground truth data. Motivated by this finding, we investigated segmentation performance on the 5 different ROIs. Table 2 shows that, except for CS-Net, the foveal region has consistently the lowest Dice score across segmentation methods. Visual inspection of our segmentations (see Supplementary Figs. S1B, S1C) reveals that incorrect detection of the boundary of the FAZ leads to important errors in FAZ area (FazE) and acircularity index (AIE) (see Table 2).

**Table 2.** Dice Score Per ROI (Superior (S), Nasal (N), Inferior (I), Temporal (T), and Foveal (F)) and FAZ Error Metrics

Method	F	S	N	I	T	FazE	AIE
Adaptive thres (AT)	0.84	0.87	0.88	0.88	0.86	14%	5%
Frangi + AT	0.79	0.86	0.86	0.85	0.85	12%	9%
Gabor + AT	0.75	0.78	0.80	0.75	0.77	13%	10%
SCIRD-TS + AT	0.71	0.73	0.75	0.67	0.71	14%	7%
OOF	0.84	0.86	0.88	0.86	0.85	24%	11%
CNN	0.82	0.84	0.85	0.84	0.83	6%	6%
U-Net	0.87	0.90	0.90	0.90	0.89	**5%**	**4%**
CS-Net	0.89	0.89	0.90	0.89	0.88	14%	5%

## Discussion and Conclusions

Retinal image analysis has demonstrated great potential for the discovery of biomarkers of eye-related disease and, more generally, systemic disease that undirectly affects the eye. Recently, OCTA imaging has enabled the visualization of the smallest capillaries in the retina without the need of a contrast agent. However, its potential for the assessment of pathologic conditions and the reproducibility of studies based on it relies on the quality of the image analysis. Automated OCTA image segmentation is an open problem in the field. In this study, we generate the first open dataset of retinal parafoveal OCTA images with associated ground truth manual segmentations. We pay special attention to segmenting the images in a reproducible way and demonstrate good inter- and intra-rater agreement. We present a comparison of state-of-the-art vessel enhancement and binarization procedures under a unified computational framework and make the source code available. By introducing two novel metrics, we evaluate segmentation quality measures to guide the identification of the algorithm that not only provides the best agreement with the manually segmented images but also achieves the best preservation of their network structure.

Our study shows that CS-Net reaches the best performances in almost all the considered evaluation metrics, suggesting this method as the best segmentation approach for parafoveal OCTA image segmentation. Interestingly, OOF achieves segmentation performances in line with the neural network architectures without the requirement of extensive manually segmented images for training purposes. Our results highlight challenges in the segmentation of the FAZ: (a) handcrafted filters suffer from noise enhancement in this region, indicating the necessity of masking that area or the use of denoising preprocessing procedures and more sophisticated binarization methods when those filters are applied and (b) disconnections in FAZ boundaries can arise from poor signal-to-noise ratio, this can affect its detection and the associated clinical metrics. Frame averaging and morphological operations can help in overcoming this issue as image preprocessing approaches.^17^ Moreover our study underlines how clinically relevant metrics used to analyze OCTA images are sensitive to the segmentation method. Results show up to 25% differences in vessel density accuracy depending on the method used, with differences up to 11% for methods with identical results in terms of pixelwise segmentation Dice and accuracy, suggesting that precaution should be taken when comparing the results of clinical studies and performing meta-analyses. Limitations of our study include the small sample size, the use of only superficial layer, and retinal scans captured by a single OCTA device. Future work will involve the investigation of the deep retinal layer and the optic nerve, and the assessment of robustness of our results across OCTA technologies from multiple manufacturers.

## Source Code and Data Availability

OCTA images and rater A (Y.G.) segmentations are available at https://doi.org/10.7488/ds/2729. Handcrafted filter code was implemented in MATLAB R2018b (Version 9.5). Python 3.6.9 was used to build ML methods. Keras library with Tensorflow backend was used to implement the CNN and U-Net, and Pytorch for CS-Net. Gudhi library was used to compute the topological metric.^42^ The source code is available at https://github.com/giaylenia/OCTA_segm_study.

## Supplementary Material

Supplement 1

Supplement 2

## References

[1] Jenkins AJ, Joglekar MV, Hardikar AA, Keech AC, O'Neal DN, Januszewski AS Biomarkers in diabetic retinopathy. *Rev Diabet Stud*. 2015; 12: 159–195. 26676667 10.1900/RDS.2015.12.159 PMC5397989

[2] Poplin R, Varadarajan AV, Blumer K, et al. Prediction of cardiovascular risk factors from retinal fundus photographs via deep learning. *Nat Biomed Eng*. 2018; 2: 2158–2164. 10.1038/s41551-018-0195-0 31015713

[3] DeBuc DC, Somfai MG, Koller A Retinal microvascular network alterations: potential biomarkers of cerebrovascular and neural diseases. *Am J Physiol Heart Circ Physiol*. 2017; 312: 201–212. 10.1152/ajpheart.00201.2016 PMC5336575 27923786

[4] Musa F, Muen WJ, Hancock R, Clark D Adverse effects of fluorescein angiography in hypertensive and elderly patients. *Acta Ophthalmol Scand*. 2006; 84: 740–742. 17083530 10.1111/j.1600-0420.2006.00728.x

[5] Yoon SP, Grewal DS, Thompson AC, et al. Retinal microvascular and neurodegenerative changes in alzheimer's disease and mild cognitive impairment compared with control participants. *Ophthalmol Retina*. 2019; 3: 489–499. 31174670 10.1016/j.oret.2019.02.002 PMC6586560

[6] Khadamy J, Aghdam K, Falavarjani K An update on optical coherence tomography angiography in diabetic retinopathy. *J Ophthalmic Vis Res*. 2018; 13: 487–510. 30479720 10.4103/jovr.jovr_57_18 PMC6210870

[7] Takase N, Nozaki M, Kato A, Ozeki H, Yoshida M, Ogura Y Enlargement of foveal avascular zone in diabetic eyes evaluated by en face optical coherence tomography angiography. *Retina*. 2015; 35: 2377–2383. 26457396 10.1097/IAE.0000000000000849

[8] Vadalà M, Castellucci M, Guarrasi G, Terrasi M, La Blasca T, Mulè G Retinal and choroidal vasculature changes associated with chronic kidney disease. *Graefes Arch Clin Exp Ophthalmol*. 2019; 257,1687–1698. 31147842 10.1007/s00417-019-04358-3

[9] Nesper PL, Roberts PK, Onishi AC, et al. Quantifying microvascular abnormalities with increasing severity of diabetic retinopathy using optical coherence tomography angiography. *Invest Ophthalmol Vis Sci*. 2017; 58: BIO307–BIO315. 29059262 10.1167/iovs.17-21787 PMC5693005

[10] Reif R, Qin J, An L, Zhi Z, Dziennis S, Wang R Quantifying optical microangiography images obtained from a spectral domain optical coherence tomography system. *Int J Biomed Imaging*. 2012; 2012: 509783. 22792084 10.1155/2012/509783 PMC3389716

[11] Alibhai AY, Moult EM, Shahzad R, et al. Quantifying microvascular changes using OCT angiography in diabetic eyes without clinical evidence of retinopathy. *Ophthalmol Retina*. 2018; 2: 418–427. 30820483 10.1016/j.oret.2017.09.011 PMC6391050

[12] Krawitz BD, Mo S, Geyman LS, et al. Acircularity index and axis ratio of the foveal avascular zone in diabetic eyes and healthy controls measured by optical coherence tomography angiography. *Vis Res*. 2017; 139: 177–186. 28212983 10.1016/j.visres.2016.09.019

[13] Onishi AC, Nesper PL, Roberts PK, Moharram GA, Chai H, Liu L, Jampol LM, Fawzi AA Importance of considering the middle capillary plexus on OCT angiography in diabetic retinopathy. *Invest Ophthalmol Vis Sci*. 2018; 59: 2167–2176. 29801151 10.1167/iovs.17-23304 PMC5915112

[14] Hwang TS, Gao SS, Liu L, et al. Automated quantification of capillary nonperfusion using optical coherence tomography angiography in diabetic retinopathy. *JAMA Ophthalmol*. 2016; 134: 367–373. 26795548 10.1001/jamaophthalmol.2015.5658 PMC4978127

[15] Kim AY, Chu Z, Shahidzadeh A, Wang RK, Puliafito CA, Kashani AH Quantifying microvascular density and morphology in diabetic retinopathy using spectraldomain optical coherence tomography angiography. *Invest Ophthalmol Vis Sci*. 2016; 57: OCT362–OCT416. 27409494 10.1167/iovs.15-18904 PMC4968771

[16] Zhang M., Hwang TS, Dongye C, Wilson DJ, Huang D, Jia Y. Automated quantification of nonperfusion in three retinal plexuses using projection resolved optical coherence tomography angiography in diabetic retinopathy. *Invest Ophthalmol Vis Sci*. 2016; 57: 5101–5106. 27699408 10.1167/iovs.16-19776 PMC5054727

[17] Schmidt TG, Linderman RE, Strampe MR, Chui TYP, Rosen RB, Carroll J The utility of frame averaging for automated algorithms in analyzing retinal vascular biomarkers in angiovue OCTA. *Transl Vis Sci Technol*. 2019; 8(1): 10. 10.1167/tvst.8.1.10 PMC6340247 30687581

[18] Jesus DA, Barbosa Breda J, Van Keer K, Rocha Sousa A, Abeg˜ao Pinto L, Stalmans I Quantitative automated circumpapillary microvascular density measurements: a new angioOCT-based methodology. *Eye*. 2019; 33(2): 320–326. 30206418 10.1038/s41433-018-0207-z PMC6367375

[19] Prentašic P, Heisler M, Mammo Z, et al. Segmentation of the foveal microvasculature using deep learning networks. *J Biomed Optics*. 2016; 21: 075008. 10.1117/1.JBO.21.7.075008 27401936

[20] Mou L, Zhao Y, Chen L, et al. CS-Net: channel and spatial attention network for curvilinear structure segmentation. In: *International Conference on Medical Image Computing and Computer-Assisted Intervention*. 2019; 721–730.

[21] Amat-Roldan I, Berzigotti A, Gilabert R, Bosch J Assessment of hepatic vascular network connectivity with automated graph analysis of dynamic contrast-enhanced us to evaluate portal hypertension in patients with cirrhosis: a pilot study1. *Radiology*. 2015; 277: 268–276. 26020435 10.1148/radiol.2015141941

[22] Alves AP, Mesquita ON, Gómez-Gardeñes J, Agero U Graph analysis of cell clusters forming vascular networks. *R Soc Open Sci*. 2018; 5: 171592. 29657767 10.1098/rsos.171592 PMC5882691

[23] Ritchie CW, Wells K, Ritchie K The PREVENT research programme—a novel research programme to identify and manage midlife risk for dementia: The conceptual framework. *Int Rev Psychiatr*. 2013; 25: 748–754. 10.3109/09540261.2013.869195 24423227

[24] Jiang H, Wei Y, Yingying S, et al. Altered macular microvasculature in mild cognitive impairment and Alzheimer disease. *J Neuroophthalmol*. 2018; 38: 1536–5166. 10.1097/WNO.0000000000000580 PMC5902666 29040211

[25] Van De Kreeke JA, Nguyen HT, Konijnenberg E, et al. Optical coherence tomography angiography in preclinical Alzheimer's disease. *Br J Ophthalmol*. 2020; 104: 157–161. 31118186 10.1136/bjophthalmol-2019-314127 PMC7025728

[26] Spaide RF, Fujimoto JG, Waheed NK Image artifacts in optical coherence tomography angiography. *RETINA*. 2015; 35: 2163–2180. 26428607 10.1097/IAE.0000000000000765 PMC4712934

[27] Linderman RE, Cava JA, Salmon AE, et al. Visual acuity and foveal structure in eyes with fragmented foveal avascular zones. *Ophthalmol Retina*. 2020; 4(5): 535–544. 31956075 10.1016/j.oret.2019.11.014 PMC7211138

[28] Yushkevich PA, Piven J, Cody Hazlett H, et al. User-guided 3D active contour segmentation of anatomical structures: significantly improved efficiency and reliability. *Neuroimage*. 2006; 31: 1116–1128. 16545965 10.1016/j.neuroimage.2006.01.015

[29] Eladawi N, Elmogy M, Helmy O, et al. Automatic blood vessels segmentation based on different retinal maps from OCTA scans. *Comp Biol Med.* 2017; 89: 150–161. 10.1016/j.compbiomed.2017.08.008 28806613

[30] Frangi AF, Niessen WJ, Vincken KL, Viergever MA Multiscale vessel enhancement filtering in Medical Image Computing and Computer-Assisted Intervention MICCAI’98, Lecture Notes in Computer Science. In: Wells WM, Delp S, Eds., Berlin, Germany: Springer Verlag, 1998; 1496: 130–137.

[31] Soares JV, Leandro JJ, Cesar RM, Jelinek HF, Cree MJ Retinal vessel segmentation using the 2-D Gabor wavelet and supervised classification. *IEEE Trans Med Imaging*. 2006; 25: 1214–1222. 16967806 10.1109/tmi.2006.879967

[32] Annunziata R, Trucco E. Accelerating Convolutional Sparse Coding for Curvilinear Structures Segmentation by Refining SCIRD-TS Filter Banks. *IEEE Trans Med Imaging*. 2016; 35: 2381–2392. 27214893 10.1109/TMI.2016.2570123

[33] Law MW, Chung AC Three dimensional curvilinear structure detection using optimally oriented flux. *European conference on computer vision*. Berlin: Springer; 2008.

[34] Bradley D, Roth G. Adaptive thresholding using the integral image. *J. Graphics Tools*, 2007; 12: 13–21.

[35] Li A, You J, Du C, Pan Y Automated segmentation and quantification of OCT angiography for tracking angiogenesis progression. *Biomed Opt Expr*. 2017; 8: 5604. 10.1364/BOE.8.005604 PMC5745106 29296491

[36] Otsu N. A threshold selection method from gray-level histograms. *IEEE Trans Systems Man Cybernetics*.1979; 9: 62–66.

[37] Rodrigues P, Guimarães P, Santos T, et al. Two-dimensional segmentation of the retinal vascular network from optical coherence tomography. *J Biomed Opt*. 2013; 18: 126011. 24343442 10.1117/1.JBO.18.12.126011

[38] McHugh ML Interrater reliability: the kappa statistic. *Biochem Med*. 2012; 3: 276–282. PMC3900052 23092060

[39] Gegundez-Arias ME, Aquino A, Bravo JM, Marin D A function for quality evaluation of retinal vessel segmentations. *IEEE Trans Med Imaging*. 2012; 31: 231–239. 21926018 10.1109/TMI.2011.2167982

[40] Hu X, Fuxin L, Samaras D, Chen C Topology-preserving deep image segmentation. In: Wallach H, Larochelle H, Beygelzimer A, d'Alché-Buc F, Fox E, Garnett R Advances in Neural Information Processing Systems. 2019;5657–5668.

[41] Schneider S, Sbalzarini IF. Finding faces in a planar embedding of a graph. Available at: http://mosaic.mpi-cbg.de/docs/Schneider2015.pdf. Accessed July 31, 2019.

[42] The GUDHI Project, GUDHI User and Reference Manual, 3.1.1 ed. GUDHI494 Editorial Board, 2020. [Online]. Available at: https://gudhi.inria.fr/doc/3.1.1. Accessed September 02, 2019.

