# Supplementary material for: Automated Segmentation of Optical Coherence Tomography Angiography Images: Benchmark Data and Clinically Relevant Metrics
Source: Transl Vis Sci Technol. 2020 Dec 3;9(13):5. doi: 10.1167/tvst.9.13.5 (PMC7718823; doi:10.1167/tvst.9.13.5)
Supplement: Supplement 1 [file tvst-9-13-5_s001.pdf]

Table S1: Table of parameters for the handcrafted filters

	Parameters	
Frangi	FrangiScaleRange	[0.5, 2]
	FrangiScaleRatio	0.5
	FrangiBetaOne	1
	FrangiBetaTwo	15
Gabor	scales	[1,2,3,4]
	epsilon	4
	k0	[0 3]
SCIRD–TS	*fb_parameters.sigma_1*	[1,5]
	fb_parameters.sigma_1_step	0.5
	fb_parameters.sigma_2	[1,2]
	fb_parameters.sigma_2_step	0.5
	fb_parameters.k	[-0.1, 0.1]
	fb_parameters.k_step	0.025
	fb_parameters.angle_step	10
	fb_parameters.filter_size	9
	alpha	0.05
OOF	range	[0.5, 2]
	sigma	0.5
	upthreshold	70

Table S2: CNN layers architecture

Layer	Type	Maps and size	Kernel size
0	Input	1 map of 61×61 neurons	
1	Convolution2D	32 maps of 56×56 neurons	6×6
2	Maxpooling2D	32 maps of 28×28 neurons	2×2
3	Convolution2D	32 maps of 24×24 neurons	5×5
4	Maxpooling2D	32 maps of 12×12 neurons	2×2
5	Convolution2D	32 maps of 9×9 neurons	4×4
6	Maxpooling2D	32 maps of 5×5 neurons	2×2
7	Dense	150 neurons	
8	Dropout		
9	Dense	1 neuron	

Table S3: U-Net layers architecture

Layer	Type	Maps and size	Kernel size
0	Input	1 map of 32×32 neurons	
1	Convolution2D	32 maps of 32×32 neurons	3×3
2	Convolution2D	32 maps of 32×32 neurons	3×3
3	Maxpooling2D	32 maps of 16×16 neurons	2×2
4	Convolution2D	64 maps of 16×16 neurons	3×3
5	Convolution2D	64 maps of 16×16 neurons	3×3
6	Maxpooling2D	64 maps of 8×8 neurons	2×2
7	Convolution2D	128 maps of 8×8 neurons	3×3
8	Convolution2D	128 maps of 8×8 neurons	3×3
9	Upsampling2D	128 maps of 16×16 neurons	2×2
10	Concatenate	192 maps of 16×16 neurons	
11	Convolution2D	64 maps of 16×16 neurons	3×3
12	Convolution2D	64 maps of 16×16 neurons	3×3
9	Upsampling2D	64 maps of 32×32 neurons	2×2
10	Concatenate	96 maps of 32×32 neurons	
11	Convolution2D	32 maps of 32×32 neurons	3×3
12	Convolution2D	32 maps of 32×32 neurons	3×3
13	Convolution2D	1 map of 32×32 neurons	3×3
