# Supplementary figures and images for: Automated Segmentation of Optical Coherence Tomography Angiography Images: Benchmark Data and Clinically Relevant Metrics
Source: Transl Vis Sci Technol. 2020 Dec 3;9(13):5. doi: 10.1167/tvst.9.13.5 (PMC7718823; doi:10.1167/tvst.9.13.5)

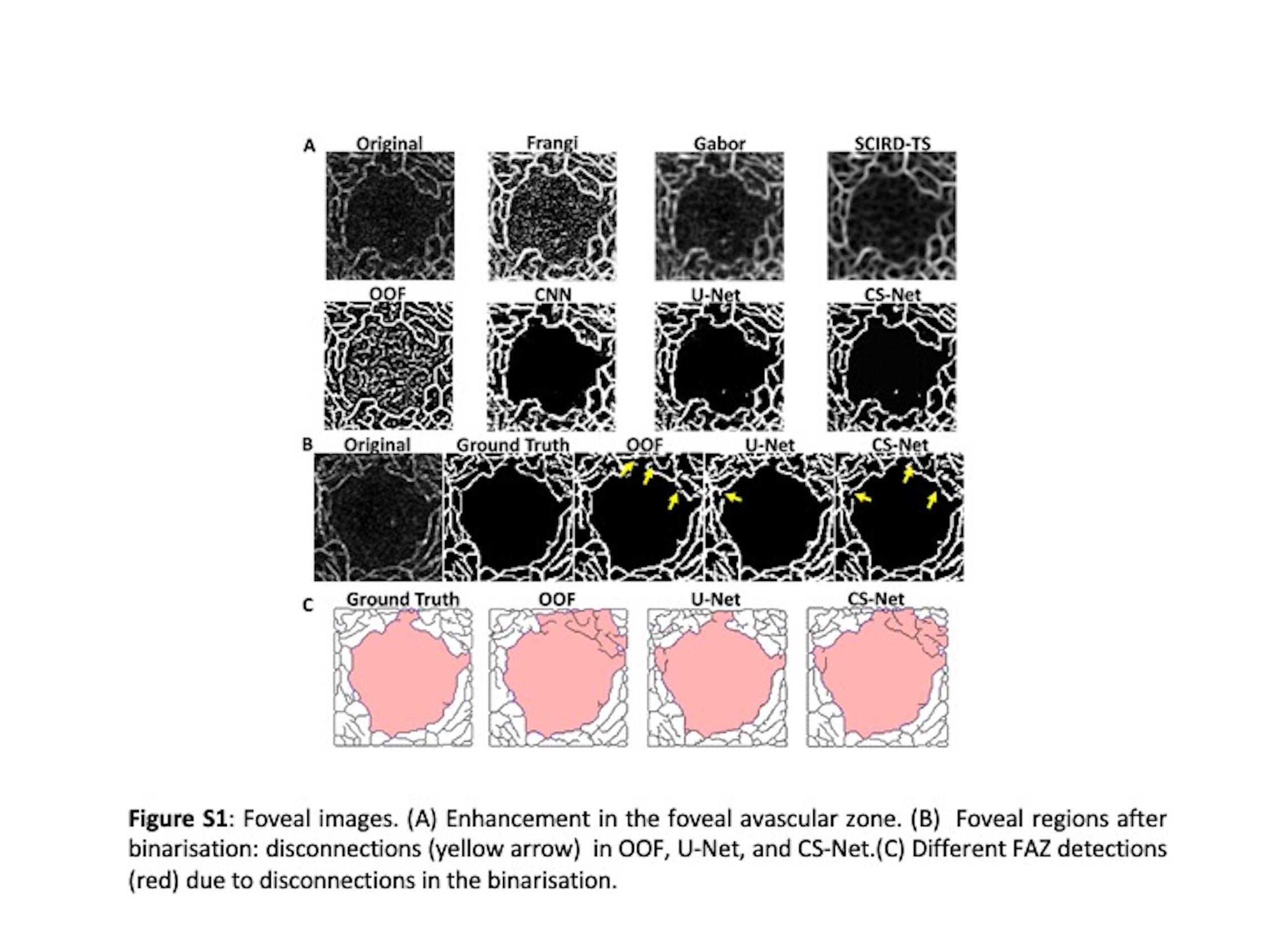

Supplement: Supplement 2 [file tvst-9-13-5_s002.jpg]
